# Human Health Risk of Organochlorine Pesticides in Foods Grown in Nigeria

**DOI:** 10.5696/2156-9614-7.15.63

**Published:** 2017-09-07

**Authors:** Aderonke O. Oyeyiola, Oluwatoyin T. Fatunsin, Latifat M. Akanbi, Damilola E. Fadahunsi, Muyideen O. Moshood

**Affiliations:** Department of Chemistry, University of Lagos, Nigeria

**Keywords:** estimated daily intake, food, health risk index, organochlorine pesticides, risk assessment

## Abstract

**Background.:**

Production of foods safe for consumption is an important issue worldwide. Organochlorine pesticides (OCP) are often used to preserve crops. Their use can have serious impacts on human health.

**Objectives.:**

This research aims at assessing OCP residues in food samples from markets in Lagos in southwestern Nigeria and their risk to human health.

**Methods.:**

Sixteen food samples were collected from Lagos, Nigeria and analyzed for organochlorine pesticide residues. Extraction was performed using an ultrasonicator, and analysis was carried out using a gas chromatograph-electron capture detector. Risk assessment was carried out by determining the hazard quotient.

**Results.:**

Results of the OCP residues found in the foods were generally low, with dichlorodiphenyldichloroethylene (p,p'-DDE) having the highest residue concentration. The concentration of p,p'-DDE was as high as 110 ng/g in pulses and 123 ng/g in Cameroon and chili peppers. The risk index was <1 in most cases, with the exception of dichlorodiphenyldichloroethane (p,p'-DDD) in fruits for children.

**Discussion.:**

The results were generally lower than what has been documented in other parts of the world and suggest that consumption of the foods investigated in the present study in Nigeria may be considered safe for the 13 OCPs investigated.

**Conclusions.:**

There is a need for continuous monitoring of these OCPs and investigation of carbamate and phosphate pesticide residues since they are more widely used in farming practices in Nigeria.

## Introduction

Pesticides are widely used in agriculture to kill unwanted organisms that may affect crops, thus improving agricultural yield, preventing pest attacks and boosting farm output. Among the pesticides most commonly used are organochlorine pesticides (OCP). These chemicals are of ecotoxicological and public health risk concern.^1^ At low concentrations, OCPs act as blockers of sex hormones, leading to changes in sexual development, abnormal sex ratios, and abnormal mating behaviors in animals. They also interfere with other hormonal processes, such as thyroid production, which influences bone development.^1^ Symptoms of pesticide exposure include headache, vomiting, skin rash, respiratory problems, and convulsions.^2^ Other potential effects of prolonged exposure to pesticides include cancer.^3^ Organochlorine pesticide residues are capable of disrupting DNA (deoxyribonucleic acid) in unborn children, the endocrine system, as well as damaging nerves and brain cells.^4^,^5^ According to the World Health Organization (WHO), pesticides have been categorized as class I (extremely hazardous) or class II (slightly toxic) according to their toxicity and have been reported to be poisonous, hazardous, and toxic to humans.^4^,^6–9^

Despite the public health risks, application of these substances to agricultural produce is on the increase in Nigeria, in order to increase food and agricultural productivity.^10^ They are applied to prevent crop damage and meet the high demand for farm produce. However, there is poor monitoring and lack of internal regulations and control by the Nigerian government.^10–12^ The WHO has reported roughly three million cases of pesticide poisoning annually, resulting in 220,000 deaths worldwide.^13^,^14^

A developing country such as Nigeria in West Africa, with a population of more than 160 million people, is economically diverse and uses pesticides in the mass production of foods and agricultural products to meet the nation's food demand. Lagos is Africa's biggest city and one of the fastest growing metropolis in the world with a population of about 20 million people. It is the most heavily industrialized city in Nigeria, where much of the nation's wealth and economic activity is located.^15^ Due to its large population, most of the food produced/grown in the north and elsewhere in Nigeria ends up in Lagos City and/or Lagos State.

**Abbreviations**
*BHC*: Benzene hexachloride
*DDT*: Dichlorodiphenyltrichloroethane
*HRI*: Health risk index
*LOD*: Limits of detection
*LOQ*: Limits of quanti3cation
*OCP*: Organochlorine pesticides
*p,p'-DDD*: Dichlorodiphenyl-dichloroethane
*p,p'-DDE*: Dichlorodiphenyl-dichloroethylene

Pesticide residues in food have been responsible for several cases of food poisoning and death in Nigeria.^16^ This is due to high levels of pesticide residue arising from improper application and multiple sprays of sub-lethal doses upon food. The incidents were investigated and several laboratory analyses conducted by the National Agency for Food and Administrative Control (NAFDAC) concluded that the deaths were caused by the consumption of poisoned beans which contained high levels of pesticides.^10^ Other incidences have been reported in Cross River State, Gombe State, and Taraba State in 2007 and 2008 by the Nigerian news.^16^,^17^

There have been studies on the presence of OCPs in some Nigerian grains. Studies by Ogah in Borno state (northern Nigeria) revealed the presence of lindane, diazinon and aldrin in pre-storage bean samples.^18^ Dichlorodiphenyltrichloroethane (DDT), dichlorvos and endrin were found in both pre- and post-storage samples.^18^ Pesticide residues in spinach, lettuce, onions, cabbage and tomatoes from Borno state were studied by Akan et al.^19^ The Akan study revealed that the amount of pesticides in the vegetables was above food tolerance levels. There was also a case of “killer” noodles in Nigeria. Children died and others were hospitalized after eating a particular brand of noodles in 2004.^20^ Investigation revealed elevated levels of pesticide residues in the dried chili peppers used in the noodles. Despite studies of pesticide levels in fruits, vegetables and grains grown in Nigeria, there has been no study assessing the risk involved and the human health impact.

The aim of this research was to quantify OCP levels in some common fruits, vegetable and grains available in local markets in Lagos State and to assess the human health risk associated with exposure. Furthermore, levels of OCPs were compared to international safety limits.

## Methods

### Reagents and Materials

OCP standards were obtained from Supelco (Bellefonte, PA, USA). Dichloromethane, ethylacetate, n-hexane and sodium sulphate anhydrous were bought from Sigma Aldrich, Inc. Germany (purity 98–99%). Solid phase extraction cartridges especially made for pesticides (Varian EnvirElut) were purchased from Varian Inc., USA.

### Sampling

Samples of watermelon (Citrullis lanatus), carrot (Daucus carota), cucumber (Cucumis sativus), cabbage (Brassica oleracea), lettuce (Lactuca sativa), wheat (Triticum spp), millet (panicium spp), sorghum (Sorghum bicolor), beans (Phaseolus spp), maize (Zea mays), tomato (Lycopersicon esculentum), chili pepper (Capsicum frutescens), Cameroon pepper (Piper nigrum), green bell pepper (Capsicum annum), Scotch Bonnet (Capsicum chinense) (local name: atarodo), and cowpea (Vigna unguiculata) (local name: white beans) were purchased from local wholesale markets in Lagos (Mile 12 Market and Oyingbo Market—two of the major markets in Lagos State) that receive these foods from northern Nigeria. The mile 12 Market is the biggest foodstuff market in Lagos State. The samples were collected and transported to the laboratory using standard sampling procedures.^21^ The samples were milled/blended, homogenized and stored at a temperature of < 4°C prior to extraction.

#### Ultrasonic Extraction and Clean-up

An aliquot of 5.0 g of each sample was weighed into a 50-ml conical flask and 2.5 g of anhydrous sodium sulphate was added and mixed with the sample to absorb any moisture present. The sodium sulphate was pretreated by heating in a furnace for 45 minutes at 550°C and stored in a desiccator. The mixture in the conical flask was extracted with 20 ml of ethyl acetate and shaken at 270 rpm for 5 minutes. The mixture was then sonicated for 20 minutes at 40°C, after which it was allowed to stand for 5 minutes and centrifuged for 5 minutes at 2500 rpm. The supernatant was concentrated to about 1 ml under a gentle stream of nitrogen gas.

The solid phase extraction cartridges were conditioned with methanol followed by distilled water and then ethyl acetate. The sample extract was loaded on the solid phase extraction column, and eluted with a solvent mixture of n-hexane: dichloromethane (3:2). The eluate was concentrated under a gentle stream of nitrogen gas and reconstituted to 1 ml using n-hexane in an amber sample vial, and taken for gas chromatograph-electron capture detector (GC-ECD) analysis.

The samples were analyzed using a gas chromatograph (Agilent 7890A) equipped with an electron capture detector. Analytes were separated with an HP 5 column (30 m × 0.25 mm × 0.25 μm). Nitrogen was used as the carrier gas and the total run time was 24 minutes.

### Method Validation

The accuracy and precision of the results were evaluated by recovery studies.

Precision was determined by analyzing the samples in triplicates. The sensitivity of the instrument was determined by calculating the limits of detection (LOD) and limits of quantification (LOQ). LOD is three times standard deviation of the blank signal, and this was calculated as the concentration at which baseline noise to signals is 3 at the expected retention time for the individual target pesticide.^22–24^ LOQ was the concentration leading to a signal-to-noise ratio of 10.^24^ For measurement values below LOQ, a value equal to the LOQ was used for statistical analyses/computation of risk assessment.

### Risk Assessment

Risk was assessed by calculating the health risk index (HRI) using Equation 1.

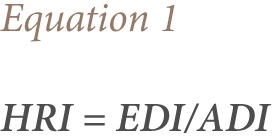



This was done based on the levels of the OCP residues found in the food samples. Estimated daily intakes (EDI) were determined and compared with the established acceptable daily intake (ADI).^25–27^ Estimated daily intake was found by multiplying the residual pesticide concentration (mg/kg^−1^) by the food consumption rate (kg/day^−1^) and dividing by body weight.^28^ Calculations were performed for adults and children (age 2–5 years). Adults were considered to have an average weight of 60 kg, while children (2–5 years) were considered to have an average body weight of 16.7 kg.^28^,^29^

Although there are different body weight estimates for children aged 6–9 and 10–12, the present study used the weight estimate of children 2–5 years of age since their lower body weight increases their exposure risk and to simplify the data analysis.

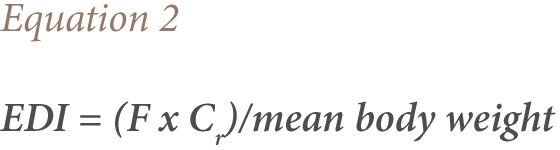
Where F = food consumption data, and C_r_ is the concentration of the residue in the food sample.

## Results

Thirteen OCPs were determined in the studied foods and are reported along with their LOD and LOQ in Tables 1 and 2. The concentration found in grains (cereals and pulses) are shown in Table 1, while Table 2 gives the results for fruits and vegetables. The results of recovery studies carried out were generally acceptable (75 and 110% recovery), and the laboratory (University of Lagos Analytical and Environmental Laboratory) is an accredited laboratory often involved in United Nations Environment Programme interlaboratory studies for organic and inorganic analytes. The relative standard deviation of replicate analysis was below 15% in most cases and less than 20% in all cases. A high relative standard deviation was found in samples with concentrations of analytes close to the detection limit.

**Table 1:** Concentration of 13 Organochlorine Pesticides in Grains (Cereals and Pulses) (ng/g)

		**Cereals**					**Pulses**					
	**Pesticide**	**Millet**	**Maize**	**Wheat**	**Sorghum**	**Mean**	**Beans**	**Cowpea**	**Mean**	**LOD**	**LOQ**_	**Recovery (%)**
1	Aldrin	<0.3	<0.3	3.45	2.34	**1.6 ± 1.6**	3.5	<0.3	**1.9 ± 2.3**	0.1	0.3	109
2	Alpha-BHC	1.25	<0.5	1.3	0.74	**0.95 ±0.39**	21	<0.5	**10.8 ± 14.5**	0.15	0.5	85.7
3	Beta-BHC	<0.75	1.69	3.12	3.09	**2.16 ± 1.2**	3.04	1.33	**2.19 ± 1.21**	0.2	0.75	96.1
4	Chlorothalonil	<0.2	<0.2	<0.2	<0.2	**0.2 ± 0.0**	<0.2	<0.2	**0.20 ± 0.00**	0.07	0.2	75.1
5	Delta-BHC	<0.03	<0.03	<0.03	<0.03	**0.03 ± 0.0**	<0.03	<0.03	**0.03 ± 0.00**	0.01	0.03	107
6	Dieldrin	<0.1	<0.1	<0.1	<0.1	**0.1 ± 0.0**	0.12	0.1	**0.11 ± 0.01**	0.04	0.1	93.4
7	Endosulfan I	<0.09	<0.09	0.09	0.09	**0.09 ± 0.00**	0.09	0.09	**0.09 ± 0.00**	0.03	0.09	96.5
8	Heptachlor	<0.3	1.38	<0.3	<0.3	**0.57 ±0.54**	<0.3	1.6	**0.95 ± 0.92**	0.1	0.3	82.3
9	Heptachlor-epoxide (B)	1.06	<0.5	0.94	1.07	**0.89 ± 0.27**	0.9	1.1	**1.0 ± 0.1**	0.15	0.5	87.5
10	Lindane	<0.2	<0.2	<0.2	<0.2	**0.2 ± 0.0**	0.31	<0.2	**0.26 ± 0.08**	0.07	0.2	98.7
11	p,p'-DDD	0.73	0.64	<0.25	<0.25	**0.47 ± 0.25**	<0.25	<0.25	**0.25 ± 0.00**	0.1	0.25	91.2
12	p,p'-DDE	<0.5	<0.5	<0.5	223	**56.1 ± 110**	106	110	**108 ± 3**	0.15	0.5	78.4
13	p,p'-DDT	<0.3	<0.3	1.22	<0.3	**0.53 ± 0.46**	0.34	0.3	**0.32 ± 0.03**	0.1	0.3	95.2
	**Sum**	**5.81**	**6.43**	**11.7**	**232**	**63.9**	**136**	**116**	**126**			

**Table 2:** Concentrations of 13 Organochlorine Pesticides in Vegetables and Fruits (ng/g)

	**Vegetables**									**Fruits**					
**Pesticide**	**Cabbage**	**Cameroon Pepper**	**Green Pepper**	**Chili pepper**	**Carrot**	**Lettuce**	**Tomato**	**Scotch Bonnet**	**Mean**	**Water melon**	**Cucumber**	**Mean**	**LOD**	**LOQ**	**Recovery (%)**
Aldrin	1.07	1.96	1.94	1.96	1.22	1.9	0.7	1.9	**1.58 ± 0.51**	1.92	<0.3	**1.11 ± 1.15**	0.1	0.3	109
Alpha-BHC	<0.5	0.82	0.73	0.67	0.58	0.82	<0.5	0.71	**0.67 ± 0.13**	0.7	<0.5	**0.6 ± 0.14**	0.15	0.5	85.7
Beta-BHC	1.64	1.8	1.72	1.78	0.86	1.75	1.66	1.76	**1.62 ± 0.31**	1.8	<0.75	**1.28 ± 0.74**	0.2	0.75	96.1
Chlorothalonil	<0.2	<0.2	<0.2	<0.2	<0.2	<0.2	<0.2	<0.2	**0.2 ± 0.0**	<0.2	0.34	**0.27 ± 0.10**	0.07	0.2	75.1
Delta-BHC	<0.03	<0.03	<0.03	<0.03	<0.03	<0.03	<0.03	<0.03	**0.03 ± 0.0**	<0.03	<0.03	**0.03 ± 0.00**	0.01	0.03	107
Dieldrin	<0.1	<0.1	<0.1	<0.1	<0.1	0<.1	<0.1	<0.1	**0.1 ± 0.0**	<0.1	<0.1	**0.1 ± 0.0**	0.04	0.1	93.4
Endosulfan I	0.17	0.29	0.27	0.29	<0.09	0.28	0.16	0.29	**0.23 ± 0.08**	0.29	<0.09	**0.19 ± 0.14**	0.03	0.09	96.5
Heptachlor	0.57	<0.3	<0.3	<0.3	<0.3	<0.3	<0.03	<0.3	**0.3 ± 0.14**	<0.3	0.73	**0.52 ± 0.30**	0.1	0.3	82.3
Heptachlor-epoxide (B)	<0.50	0.58	<0.5	0.58	<0.50	<0.50	<0.50	<0.50	**0.52 ± 0.04**	0.56	<0.50	**0.53 ± 0.05**	0.15	0.5	87.5
Lindane	0.22	0.23	<0.2	0.21	<0.2	<0.2	<0.2	<0.2	**0.21 ± 0.01**	<0.2	<0.2	**0.2 ± 0.0**	0.07	0.2	98.7
p,p'-DDD	<0.25	0.38	<0.25	0.38	<0.25	0.26	<0.25	0.29	**0.29 ± 0.06**	0.37	<0.25	**0.31 ± 0.08**	0.1	0.25	91.2
p,p'-DDE	86.1	123	11.9	123	115	119	10.2	119	**88.5 ± 49.2**	123	41.7	**82.4 ± 57.5**	0.15	0.5	78.4
p,p'-DDT	<0.3	0.49	<0.3	<0.3	<0.3	<0.3	<0.3	<0.3	**0.32 ± 0.07**	<0.3	<0.3	**0.3 ± 0.0**	0.1	0.3	91.2
**Sum**	**91.7**	**130**	**18.4**	**131**	**120**	**126**	**14.8**	**126**	**94.6**	**130**	**45.8**	**87.8**			

Concentrations of the various OCPs varied from below their LOQ to high levels. In cereals, α- benzene hexachloride (BHC) had concentrations between <0.5 and 1.25 ng/g, while beans and cowpeas had concentrations of 21 and <0.5 ng/g, respectively. The highest concentration of β-BHC in the present study was found in wheat (3.12 ng/g), followed by sorghum (3.09 ng/g) and then beans (3.04 ng/g). The lindane concentration for most of the grain samples was below the LOQ values (0.2 ng/g) with the exception of beans, which had a concentration of 0.31 ng/g. Chlorothalonil, δ-BHC and heptachlor all had concentrations below their LOQ except for cowpea, which had a concentration of 1.6 ng/g of heptachlor. The concentration of aldrin in cereals ranged between < 0.3 ng/g (millet and maize) and 3.45 ng/g (wheat), while the pulses, (beans in this case), had a high concentration of 3.5 ng/g. Sorghum had a very high concentration of dichlorodiphenyldichloroethylene (p,p'-DDE) (233 ng/g), although the level in other cereals was below their LOQs of 0.5 ng/g. The pulses also had levels of p,p'-DDE as high as 106 ng/g and 110 ng/g for beans and cowpeas, respectively.

Concentrations of the 13 OCPs determined in fruits and vegetables are shown in Table 2. Alpha–BHC in vegetables had concentrations between <0.5 and 0.73 ng/g, while watermelon and cucumber (fruits) had concentrations of 0.7 and <0.5 ng/g, respectively. Concentrations of beta-BHC for vegetables and fruits in this study were between <0.75 and 1.8 ng/g. Lindane concentrations for vegetables and fruits were relatively low, and varied between <0.2 and 0.23 ng/g. Chlorothalonil, delta-BHC and heptachlor all had concentrations below their LOD with the exception of cucumber, which had a concentration of 0.34 ng/g of chlorothalonil. Cabbage and cucumber had concentrations of 0.57 and 0.73 ng/g, for heptachlor, respectively. The concentration of aldrin in vegetables ranged between 0.7 (tomatoes) and 1.96 ng/g (Cameroon pepper), while in watermelon and cucumber the concentrations were 1.92 and <0.3 ng/g, respectively. The concentration range for p,p'-DDE in vegetables was between 10.2 and 123 ng/g, while in the two fruits studied, watermelon and cucumber had concentrations of 123 and 41.7 ng/g, respectively. The calculated risk indices were less than 1 for adults in all cases and less than 1 for children in most cases, with the exception of p,p'-DDE.

## Discussion

Generally, the levels of the 13 OCPs determined were found to be lower in vegetables and fruits than in grains. Hlihor et al. determined pesticide residues in yellow peppers (a vegetable similar to chili and green pepper) and found the concentrations of chlorothalonil residue in the range of 1140 to 2750 ng/g.^30^ Endosulfan in the study by Latifet al. was in the range of < 150 to 1330 ng/g for green chili (called green pepper in this study), <150 – 580 ng/g for tomatoes.^8^ Lozowicka et al determined OCPs residues in black currant, beans, carrots, celery, cucumbers, lupine, parsley, tomatoes and cereals collected from Kazakhstan and Poland and found that these fruits, vegetables, and cereals contained residues of different OCPs including aldrin, dieldrin, hexachlorocyclohexane isomers and DDT metabolites (p,p'-DDE and dichlorodiphenyldichloroethane (p,p'-DDD)).^31^ Endosulfan and dicofol were also found in some samples in concentrations ranging from 8.0 to 800 ng/g. DDT and its metabolites were also detected. In another study of OCPs in cowpeas from markets in Ile-Ife, southwestern Nigeria, the concentration ranges for alpha-, beta-, and delta-BHC were between 7 to 44 ng/g, 11 to 144 ng/g and 19 to 125 ng/g, respectively.^32^ In another study in Lagos, Nigeria, the mean concentration of aldrin, DDT, dieldrin, endosulfan and endrin residues in beans was 9.8, 35.1, 5.8, 22.5 and 7.8 ng/g, respectively.^33^ Based on this and other studies conducted in Nigeria on the levels of OCPs in foods, concentrations of OCPs were lower than those reported from other parts of the world. In addition, the OCP levels found in this study were lower than those reported in previous studies from Nigeria. This may be a result of improved monitoring by regulatory agencies or due to increased use of organophosphate and organocarbamate pesticides in Nigeria. The list of commonly used pesticides in Nigeria is dominated primarily by carbamates and organophosphates, and only a few organochlorine pesticides such as lindane, DDT, heptachlor and aldrin were mentioned by Ita.^34^ Secondly, organic farming where little or no chemicals are used in farming, is still being practiced by subsistence farmers, who make up a large proportion of farmers in Nigeria.

It is important to assess the risks associated with dietary exposure to pesticides in order to protect the health of consumers.^35^ Since maximum residual limits are not toxicological limits, the levels of exposure to OCPs found in this study were compared with the acceptable daily intake as established by the Food and Agriculture Organization of the United Nations/World Health Organization (FAO/WHO) food standard program Joint Meeting on Pesticide Residue and the Australian government to estimate the human health risk from consumption of foodstuffs.^26^,^36^ The foods sampled were divided into cereals, pulses, fruits and vegetables for adults, and grains, fruits and vegetables for children (aged 2–5 years). Table 3 presents the data for food consumption of major food groups (g/person/day) for children and adults in Nigeria (Diet Cluster J).^36^ Table 4 and 5 give the calculated HRI for children and adults, respectively. An index of greater than 1 indicates that consumption is unsafe for human health.^2^

**Table 3:** Consumption of Major Food Groups

**Food**	**Mean value(g/person/day)**
Ages 3–5 (United States Environmental Protection Agency)^28^	
Grains	190
Vegetables	140
Fruits	210
Adults in Nigeria (World Health Organization)^36^	
Cereals	409.7
Pulses	24.1
Vegetables	89.3
Fruits	106.9

**Table 4:**
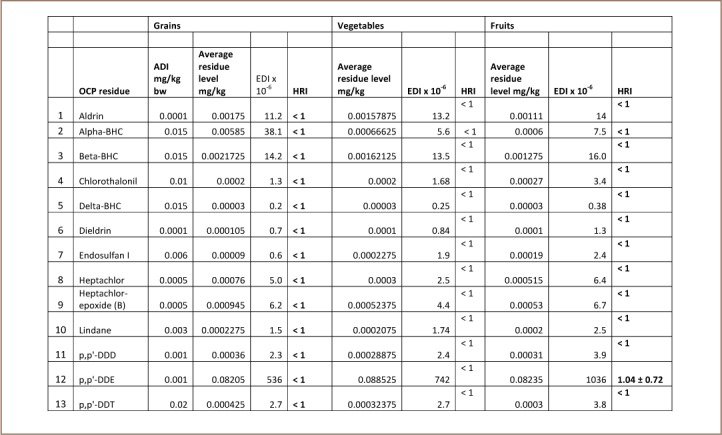
Health Risk Assessment of Pesticide Residues in Studied Foods for Children

**Table 5:**
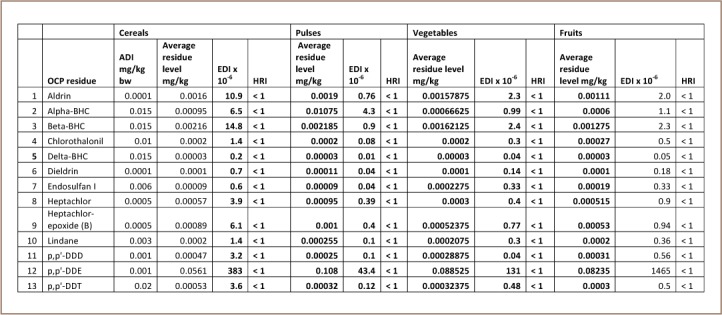
Health Risk Assessment of Pesticide Residues in Studied Foods for Adults

It was observed that the HRI was <1 in almost all cases, with the exception of p,p'-DDD in fruits when consumed by children, which had an health risk index of 1.04. In determining the health risk indices, average values were used. Using the lower end of the concentration which is the concentration in cucumber, the HRI value was far less than 1 and the standard deviation for the risk index of 1.04 was 0.72, indicating that the value was not significant. The risk index of 1.04 for fruit consumption by children may also be due to their low body weight. There is therefore a need for close monitoring of this particular residue in fruits consumed by children. For adults, the HRI was observed to be <1 in all the cases and the foods studied are considered to pose insignificant risk. This may indicate that the OCP residues found in food in the present study pose minimal human health risk.

## Conclusions

The OCP residues found in the foods investigated show that the concentrations were lower than in other parts of the world. Also, the concentrations were in most cases found to be below the maximum residual limits. This is probably a result of strict and regular monitoring of this pesticide in food since the incidence of the ‘killer noodles.’^20^ The HRIs in almost all of the cases were below 1, indicating that OCPs in foods from Lagos markets pose little or no risk, and this survey shows that OCP pesticides may no longer be used in Nigeria in the preservation of foods or control of pests. Nevertheless, monitoring programs and risk assessment studies for other pesticide residues such as carbamates and organophosphates in the foods investigated are needed.
